# Pulmonary rehabilitation integrated coached exercise training for patients with COPD: a study protocol for a randomized controlled trial

**DOI:** 10.1186/s13063-022-07058-2

**Published:** 2023-01-30

**Authors:** Yuting Jing, Yuying Ma, Hongxing Zhang, Zhenhu Wu, Yongwen Li, Haoxuan Li, Minling Huang, Lin Lin, Yinji Xu

**Affiliations:** 1 Department of Pulmonary Critical Care Medicine, Guangdong Provincial Hospital of Chinese Medicine, Guangzhou, 510120 Guangdong Province China; 2 The Second Clinical Medical College, Guangzhou University of Traditional Chinese Medicine, Guangzhou, 510403 Guangdong Province China; 3 College of Health, Guangdong Pharmaceutical University, Guangzhou, 510006 Guangdong Province China

**Keywords:** Pulmonary rehabilitation, Chronic obstructive pulmonary disease, Exercise training, Sports, Integration, Randomized controlled trial

## Abstract

**Background:**

Chronic obstructive pulmonary disease (COPD) is the most common chronic lung disease creating an immense burden on social health care systems. Pulmonary rehabilitation (PR) has proven to be effective in patients with COPD. However, exercise training as the basis of PR becomes extremely tedious, occasionally causing a loss of perseverance in patients. Therefore, we considered an approach that makes this technique interesting and easier to persist. The aim of this project was to explore an exercise training approach based on PR-integrated coached exercise training to promote the new exercise training approach as a form of group rehabilitation activity in the future.

**Methods:**

Participants will be randomly divided into the trial and control groups. The trial group will be treated with PR-integrated coached exercise training (plus usual care). All exercise programs will be guided by sports coaches with a physical education background. Meanwhile, the control group will receive traditional PR and home exercises, including walking and swimming. The study will last for 12 weeks. The primary outcome measure is exercise tolerance using the 6-min walking test and secondary outcomes are the peak oxygen uptake of cardiopulmonary exercise tests, the COPD Assessment Test, and the St. Georges Respiratory Questionnaire. Other evaluated outcomes include changes in postbronchodilator forced expiratory volume at 1^st^ second, forced vital capacity, body fat and muscle composition, and mental status measured using the Hamilton Anxiety and Depression Scales.

**Discussion:**

This study provides a simple, feasible, repeatable, and fun exercise training approach. To the best of our knowledge, there are no randomized controlled trials in the existing literature on PR-integrated coached exercise. The protocol shared in our study can be used as a reference for exercise training in patients with COPD.

**Trial registration:**

Ethical approval (BF2020-236–02) was obtained from the Guangdong Provincial Hospital of Chinese Medicine Human Research Ethics Committee. All participants signed an informed consent form. ChiCTR-2100043543. The registration date is 2021/02/21 and it is the third version.

**Supplementary Information:**

The online version contains supplementary material available at 10.1186/s13063-022-07058-2.

## Introduction

Chronic obstructive pulmonary disease (COPD) is a common, preventable, and treatable disease, characterized by persistent respiratory symptoms and airflow limitation. Airway obstruction leads to continual dyspnea, cough, sputum production, and systemic effects [1]. In 2010, the global prevalence of COPD was estimated at 11.7%, with an overall increasing trend [2]. According to the World Health Organization, COPD will become the fifth largest disease burden and the third largest cause of death by 2020 [3–5].

Pulmonary rehabilitation (PR) has been shown to be an effective therapeutic strategy to improve shortness of breath, health status, and exercise tolerance in COPD and is an important part of integrated patient management [6]. Supervised maintenance exercise training at least twice a week forms the foundation of PR and encompasses endurance training, interval training, resistance or strength training, flexibility, and inspiratory muscle training. All interventions are aimed at maximizing personal functional gains [7]. Due to individual differences, development of a uniform exercise training program becomes impossible. Walking is the most common form of training and is recommended at a constant speed of 80–120 steps per min, at least 45 min each time, to achieve a heart rate in the target range and maintain it for more than 10 min [8]. The cycle ergometer is a frequent choice for endurance training.

Available evidence indicates that optimum benefits are achieved from programs lasting for 6–8 weeks [7]. However, there are numerous challenges faced along the way and exercise training occasionally becomes too monotonous to sustain for some patients. When the program is unsupervised, patients fail to effectively carry on with the exercise training. Walking exercises without supervision cannot achieve the targeted intensity [9]. Additionally, only a minority of patients favor cycling. A study in China showed that only 3.8% of 80 patients approved of cycling [10].

Over the past few years, efforts have been made to provide more integrated, patient-centered PR programs, and engagement in sports has been suggested as a convenient setting for health promotion [11]. There are sports clubs that organize and provide opportunities for competition and participation in community sports. And also, researchers found that PR program supplemented with virtual reality techniques is beneficial intervention to improve physical fitness in patients with COPD [12]. In China, traditional Chinese medicine exercises such as Baduanjin and Tai Chi are popular and are proved to be effective exercise training [13, 14].

The integration of medicine into sports training was first proposed in physical education to prevent diseases [15]. Sports coaches focus on helping patients by conducting exercise training in an effective and interesting manner. Therefore, PR-integrated with coached exercise training may be a viable approach that offers a patient-tailored, individualized intervention, targeting long-term adherence to health-enhancing behaviors.

Considering the role of a sports coach in maintaining the efficiency and persistence of exercise training, we invited a sports coach to the PR team and designed a randomized, controlled clinical trial. The aim of the project was to explore an exercise training pattern based on PR integrated with coached exercise training.

## Methods and analysis

### Study setting

**Fig. 1:**
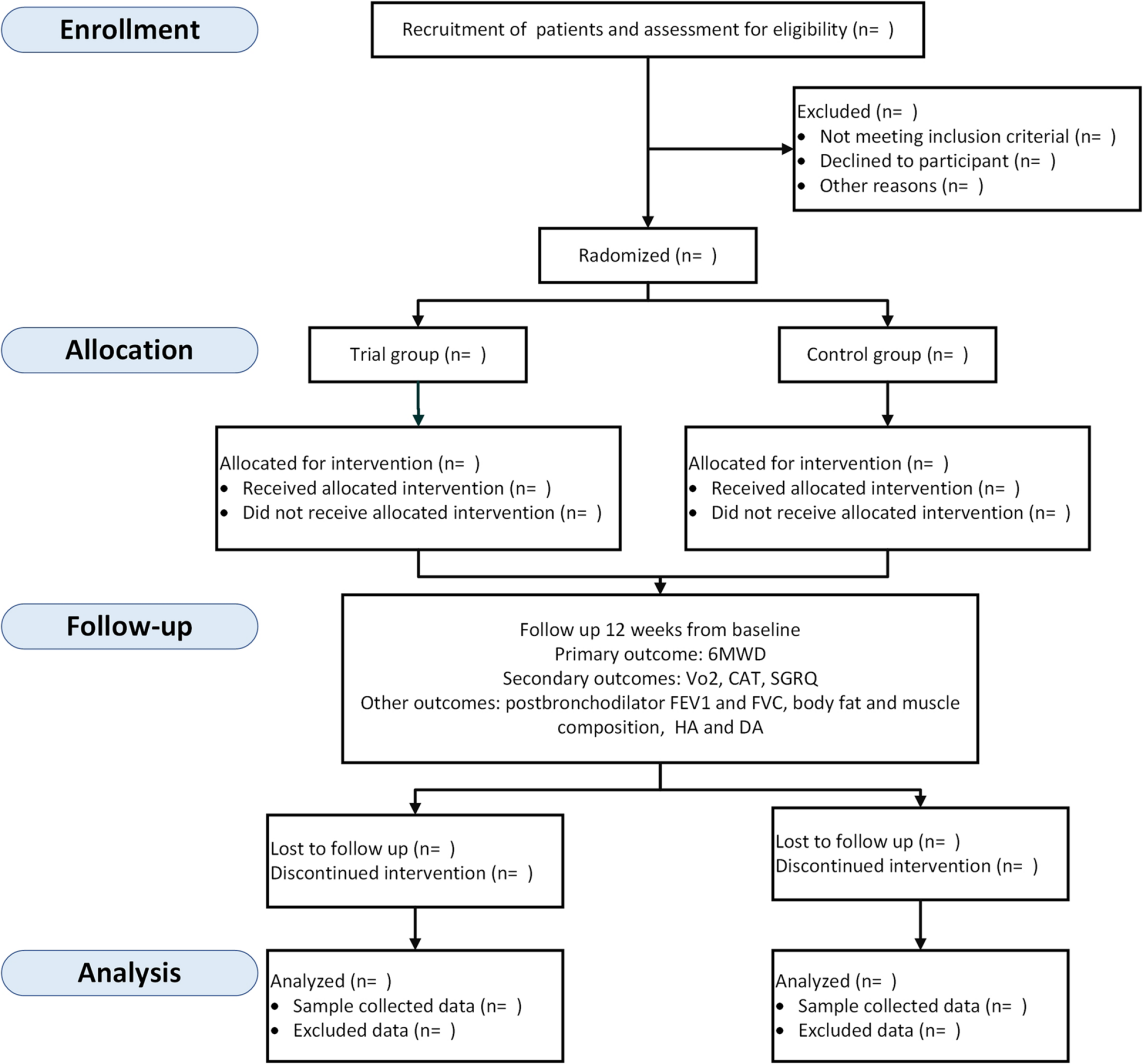
Flow chart describing study design

This study is a prospective, parallel-group randomized, controlled, exploratory clinical trial. Participants will be recruited from the Guangdong Provincial Hospital of Chinese Medicine Clinic. They are outpatients attending a clinic or inpatients being discharged home and evaluated by two experienced respiratory physicians. Patients are invited to meet with the research physicians to discuss any remaining questions and sign the informed consent. Thereafter, the participants are randomly assigned to either the trial or control group as a ratio of 1:1. The trial group will receive PR integrated with coached exercise training. The control group will receive a traditional PR program. Owing to the exercise intervention, it is not possible to blind participants or those involved in the provision of care. However, the researchers collecting primary data and performing statistical analyses will be blinded to the allocation. The design is open label with only outcome assessors and data analysts being blinded, so unblinding will not occur. The study design is shown in Fig. 1.

### Eligibility criteria

We will include participants aged between 40 and 80 years, diagnosed with COPD (a post-bronchodilator FEV1 < 70% and < 80% of predicted normal values), who are clinically stable, have not experienced an acute exacerbation for at least 4 weeks before the trial, do not participate in systematic exercise training in the past 6 months, and have a 6-min walk test (6MWT) distance between 350 and 550 m.

We will exclude participants with severe comorbidities, including coronary heart disease, arterial aneurysm, severe hepatic and renal dysfunction, and uncontrolled hypertension. Patients with mental diseases, deafness, limb activity disorder, and inability to cooperate are also excluded from the trial.

Patients can leave the study at any time for any reason. Intervention can also be ended by the investigators if the patient is uncooperative and does not attend study visits. This study will be ended in case of any abundance in adverse events or procedure-related complications.

### Randomization

A list of random numbers is generated using IBM SPSS statistical software (version 23.0). Hence, every participant will match a number and the information is blind to other trialists. Opaque envelopes containing a number are used to randomly assign participants to either the trial or control group. Two trialists will generate the allocation sequence and assign participants to interventions together.

### Sample size calculation

For the primary outcome, a change of 25 m in the 6-MWT distance is considered to be the minimal important difference (MID) in patients with COPD [16], based on a two-sample independent *t*-test with a given MID of 25 m, standard deviation of 44.6 m, power of 80%, and significance level of 0.05 [17]. Accordingly, the calculated sample size of each group is 50. Assuming a dropout rate of 15% resulted in 18 patients being included in the final study population.

### Interventions

After baseline data collection, the patients will be randomly divided into the trial or control group. Participants in both the groups will receive usual care, and PR included health education, nutrition guidance, psychological support, and exercise training [6, 18]. The intervention will be performed three times per week for 12 consecutive weeks. The only difference in intervention between both groups is the exercise training.

In the control group, exercise sessions, which mostly included walking and swimming, lasted for at least 30 min, three times a week, with a weekly follow-up. In contrast, patients in the trial group will receive exercise training guided by a sports coach, which lasts for 60 min, once a week at the hospital, and for 30 min, twice a week at home, conducted through a video. Exercise training for the trial group consists of warm-up exercise, aerobic exercise, resistance exercise, respiratory rhythm adjustment, and respiratory muscle training.

#### Warm-up exercise

Warm-up exercise is based on *Baduanjin* combined with lip-constricted breathing and abdominal breathing. *Baduanjin* is a traditional Chinese physical exercise that involves mild exercise and respiratory regulation [19].

#### Aerobic exercise

Considering the tolerance of patients with COPD, sports coaches integrate aerobic exercise into a set of aerobics. Aerobics include eight movements and could be performed in the standing or sitting position according to the patient’s condition. Each movement is performed in 4–6 sets of 8–16 repetitions. Between each set, there is a break of 10 s. If the patient is unable to tolerate the exercise, the break time could be extended.

#### Resistance training

Resistance training could also be performed while standing or sitting. The sports coach integrates resistance exercises into a set of aerobics, including eight movements with a stretch belt (1.5 m, 22 pounds). Each movement consists of 4–6 sets of 8–16 repetitions. Between each set, there is a break for 10 s. If the patient is unable to keep up, the break time will be extended.

#### Respiratory muscle training

Patients receive respiratory muscle training through abdominal breathing, wherein the abdomen gently puffed-up during inhalation and sank during exhalation. A sandbag weighing 5 kg will be placed on the abdomen, followed by abdominal breathing for 3 min.

#### Respiratory rhythm adjustment

Patients breath is through the nose and breath out through the mouth slowly. Respiratory rhythm adjustment runs through the training.

In both groups, each patient has an exercise log, which is completed by a supervisor who instructed the sessions online. The exercise log contains adverse events, completed exercises, and a record of vital signs before and after exercise.

### Outcome measures and follow-up

Data on the outcomes will be collected at baseline and after 12 weeks. Patients are followed up weekly in the outpatient department or through online methods, including telephone and WeChat. The primary outcome measure is exercise tolerance using the 6-MWT. Secondary outcomes are the peak oxygen uptake (V̇O_2_) of cardiopulmonary exercise tests, the COPD Assessment Test (CAT), and the St. Georges Respiratory Questionnaire (SGRQ). Other outcomes include changes in postbronchodilator forced expiratory volume at 1^st^ second (FEV1), forced vital capacity (FVC), body fat and muscle composition, mental status measured using the Hamilton rating scale for depression scores (HAMD-24) and the Hamilton Anxiety rating scale (HAMA).

The investigator will inquire about the occurrence of adverse events. The details of every adverse event will be reported in the case report form (CRF). The same investigator will record all outcome measures to maintain standardization in the procedure.

### Measurements

#### 6-min walking test

This test measures the distance a participant can walk in 6 min. The patients will be instructed to walk as far as possible in 6 min and receive recommended standardized encouragement. Two tests will be performed on each occasion, and the best distance is recorded. A 30-min rest will be mandatory between the first and second 6-MWT [20, 21].

#### Peak oxygen uptake of cardiopulmonary exercise tests

Cardiopulmonary exercise testing (CPET) will start with an initial rest of 3 min, followed by unloaded cycling for 3 min and a subsequent increment of 5–15 W after each minute. The aim is achieving a total exercise performance time of 8 to 12 min. Patients are asked to maintain a pedaling frequency of 50–60 rpm on a cycle ergometer (SCHILLER CS-200 Ergo- Spiro, Switzerland). If the patients display symptoms such as unsustainable dyspnea or leg fatigue, chest pain, ECG-significant ST-segment depression, and a drop in systolic blood pressure or oxygen saturation (SpO_2_) ≤ 84%, the test will be stopped. V̇O_2_ will be recorded as the mean value of VO_2_ during the last 20 s of the test. VO_2_ will be expressed both as an absolute value (l/min) and in terms of mL/kg/min [22, 23].

#### COPD Assessment Test (CAT)

CAT is a patient completes an 8-item questionnaire that assesses the impact of COPD on self-reported health status and symptoms [24]. Each item is scored from 0 to 5 points (0 indicating no impact or symptoms and 5 indicating the worst possible impact or symptoms) summing up to a total CAT score in the range of 0–40 points.

#### St. Georges Respiratory Questionnaire

The SGRQ is a self-administered questionnaire designed to measure self-perceived impairments in health and quality of life in individuals with airway diseases [25]. The three component scores of the questionnaire, including symptoms, activity, and impact (on daily life) were calculated, giving a total score between 0 and 100, wherein 0 indicates good health and 100 indicates very poor health.

#### Pulmonary function testing

Pulmonary function tests are performed by spirometry using the same machine and the same technician for all patients according to international recommendations [26, 27]. A flow-sensing spirometer and a body plethysmograph connected to a computer (Master Screen Diffusion Combined Pulmonary Function Tester, Jaeger, Germany) will be used for the measurements.

#### Body fat and muscle composition

Body fat and muscle composition will be measured using multifrequency bioelectrical impedance analysis (BIA) via a body composition analyzer (InBody 770, Biospace Co Ltd, Seoul, South Korea). The participant stands barefoot on the platform of the device with the soles of their feet on the electrodes. The participants then grasp the handles of the unit with their thumb and fingers to maintain direct contact with the electrodes and stood still for 1 min while maintaining their elbows at full extension and shoulder joints abducted to approximately 30° angle. During the assessment, BIA analyzers introduce a small electrical current into the body and measure the resistance or impedance to the current flow to calculate skeletal muscle, fat content, and other components of the body. Data on total body composition, body fat percentage, muscle mass, and bone mass will also be collected.

#### Hamilton Rating Scale for Depression (HAMD-24) and Hamilton Anxiety rating (HAMA) scales

HAMD-24 and HAMA are used to evaluate depression and anxiety levels of participants [28]. The HAMD scale includes 24 problem items, which can be classified into seven-factor structures: anxiety/somatization, body mass, cognitive impairment, day-night change, block, sleep disturbance, and sense of hopelessness. A total score of ≥ 8 indicates that the patient has positive depressive symptoms. The HAMA scale contains 14 items, most of which can be scored on a 0–4 scale.

### Participant timeline

The study will last for 3 years. Recruitment of patients started in March 2021. Baseline information including sex, age, body mass index, and history of smoking will be collected at the beginning. Vital signs including blood pressure, heart rate, oxygen saturation, and respiratory rate are collected every week during the period of intervention to evaluate safety. Outcome data are collected before intervention and at 12 weeks. The protocol follows the Standard Protocol Items: Recommendations for Interventional Trials (SPIRIT) 2013 [29]. And A brief SPIRIT flow diagram is shown in Table 1. A populated SPIRIT checklist is provided in Additional file.

**Table 1:** Tabulated summary of the study schedule

Timepoint	Baseline (t0)	Week 1–12 (t1)	Week 13 (t2)
Enrollment:			
Eligibility screen	** × **		
Informed consent	** × **		
Demographics	** × **		
Medical history	** × **		
Interventions:			
Trial group		** × **	
Control group		** × **	
Assessment:			
6-MWT	** × **		** × **
V̇O_2_ of CET	** × **		** × **
CAT	** × **		** × **
SGRQ	** × **		** × **
Pulmonary function testing	** × **		** × **
Body fat and muscle composition	** × **		** × **
HAMD-24	** × **		** × **
HAMA	** × **		** × **
Safety monitoring:			
Vital signs	** × **	×	** × **
Adverse event reporting		** × **	** × **

### Adverse event reporting

Adverse events will be recorded in CRF (Case Report Form). Serious adverse events will be reported within 24 h to the principal investigation and Institutional Review Board. The steering committee consists of a pulmonologist and a respiratory nurse who will survey the study procedure and evaluate serious adverse events. The steering group is the trialists of this study. If there is any damage related to the study, the research group will pay the medical expenses and make corresponding financial compensation according to laws and regulations.

### Data management and statistical analysis

Researchers will make appointments for the next follow-up to promote participant retention. Data will be collected in the CRF on paper by the same investigator. All the CRF on paper will be stored in a locked cabinet. Access to the data sets is only available to the investigators in this study. Incomplete data of patients who are lost to follow-up for various reasons will be eliminated. Ethics Committee of Guangdong Provincial Hospital of Chinese Medicine makes a visit per year and checks the presence and completeness of the investigation file. All substantial amendments will be notified to the Committee, and non-substantial amendments will be recorded.

Outcome measures will be analyzed using a *t*-test at the end of the trial. IBM SPSS statistical software for Windows (Version 23.0) is used to analyze the data for the outcome measures. Data is presented as the mean and standard deviation.

### Dissemination

Results of this research will be disclosed completely in international peer-reviewed journals. Both positive and negative results will be reported.

## Discussion

Many previous studies have explored ways to increase adherence to the ongoing exercise training. Some researchers integrated virtual reality into PR and suggested that a PR program supplemented with VR is a beneficial intervention to improve physical fitness in patients with COPD [12]. Home-based telerehabilitation via real-time videoconferencing has been proven to improve endurance, exercise capacity, and self-efficacy in patients with COPD [30]. Additionally, modifications of traditional Chinese medicine exercise training methods, including Baduanjin and Taiji, have been used as appropriate substitutes for PR [14, 31].

In this study, we incorporated exercise training into aerobic dancing because this is an interesting approach to exercise, which makes it easier for patients to adhere to the program. This study integrates sports coaches into the PR team, thereby providing a simple, feasible, repeatable, and fun exercise training approach. To the best of our knowledge, there are no randomized controlled trials in the existing literature on PR-integrated coached exercise training as sets of aerobics and can be performed as a group activity. The protocol shared in our study can be used as a reference for exercise training in patients with COPD. We hope that video materials for exercise training can be made available in the future to promote community- and group-focused rehabilitation activity.

This study has a few limitations. First, patients with mild or very severe COPD were not included. Second, the findings were limited because of the single-center design of the study. Finally, patient and clinician blinding were not possible because of the nature of the intervention.

## Trial status

This is the third and definitive protocol version. Participants will be recruited between March 1st, 2021, and November 31, 2023. Study completion is expected to be in June 2024. The study protocol has been submitted before the end of the recruitment and before the last patient.

## Supplementary Information

**Additional file 1.** SPIRIT Checklist for Trials

## Data Availability

The datasets analyzed during the current study are available from the corresponding author on reasonable request. The data will be available after the main publication of them; for other circumstances, they should consult the corresponding author. Any data required to support the protocol can be supplied on request.

## Abbreviations

COPD: Chronic obstructive pulmonary disease
PR: Pulmonary rehabilitation
6-MWT: 6-Min walking test
MID: Minimal important difference
V̇O2: Peak oxygen uptake
CAT: COPD Assessment Test
SGRQ: St. Georges Respiratory Questionnaire
FEV1: Forced expiratory volume at 1st second
FVC: Forced vital capacity
HAMD: Hamilton rating scale for depression scores
HAMA: Hamilton Anxiety rating scale
CRF: Case report form
CPET: Cardiopulmonary exercise testing
SpO2: Oxygen saturation
BIA: Bioelectrical impedance analysis
